# Supplementary material for: Brief interactive lifestyle preventive medicine video education in the primary care clinic: Protocol for a randomized clinical trial
Source: PLoS One. 2026 Mar 26;21(3):e0322244. doi: 10.1371/journal.pone.0322244 (PMC13020839; doi:10.1371/journal.pone.0322244)
Supplement: S1 Appendix — Data collection fields. (DOCX) [file pone.0322244.s001.docx]

**S1 appendix A: data collection fields**

REDCap will be used to collect all data

- Medical Record Number
- Age
- Sex
- Gender
- Race
- Ethnicity
- Smoking Status and use of tobacco products
- BMI
- Charlson Comorbidity Index
- Insurance Status
- Social Influencers of Health Questionnaire (if available)
- Patient Zip Code
- Insurance Status
- Follow-up questionnaire items: see page 21, S3 Appendix C for questions included during telephonic and EHR follow-up.
- If the patient is living in an institution or living independently
- HIV will not be evaluated outside of its inclusion in the aggregate Charlson Comorbidity Index (CCI), which will prevent any possibility of calculating HIV status indirectly).
