# Supplementary material for: Brief interactive lifestyle preventive medicine video education in the primary care clinic: Protocol for a randomized clinical trial
Source: PLoS One. 2026 Mar 26;21(3):e0322244. doi: 10.1371/journal.pone.0322244 (PMC13020839; doi:10.1371/journal.pone.0322244)
Supplement: S2 Appendix — Primary care clinic questionnaires and Script. (DOCX) [file pone.0322244.s002.docx]

**S2 appendix B: Primary Care Clinic questionnaires and script**

Hello! My name is (Research assistant/associate name), and I am a research associate here at the clinic. You (or a family member) came to our primary clinic today. To participate in our study, you will be asked to complete a brief questionnaire about your lifestyle habits, such as sleep, smoking, diet, and exercise. We hope that your participation will help us do a better job of giving patients instructions before they leave the clinic. Would that be okay?

DO NOT PROCEED IF INFORMED CONSENT IS NOT OBTAINED.

If informed consent is obtained, proceed with randomization of video or no video.

If the patient is randomized to video, tell the patient: “Your provider (optional - give the name of provider here) would like you to watch a video that will teach you about how to live a healthy lifestyle. Would you like to watch it now?”

NOTE: The following two questions will be administered using the REDCap data collection tool. For each prompt, the participant will be asked about their readiness score and then about their confidence level immediately after, using the corresponding visual analog scale (VAS).


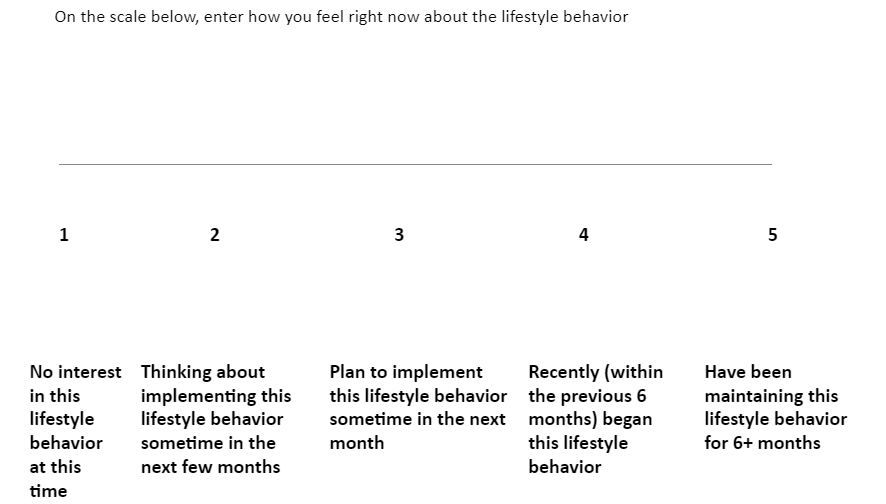


Source: Gillespie and Lenz 2011

Readiness-to-Change Lifestyle Questionnaire

Name: _______________________________

Date: ________________________________

Rating Readiness-to-Change (USE “READINESS RULER” ABOVE)

5 No interest in this lifestyle behavior at this time

4 Thinking about implementing this lifestyle behavior sometime in the next few months

3 Plan to implement this lifestyle behavior sometime in the next month

2 Recently (within the previous 6 months) began this lifestyle behavior

1 Have been maintaining this lifestyle behavior for 6+ months

Rating Lifestyle behavior

______ Purposeful exercise at least 5 times per week

______ Incorporate “extra” physical activity throughout the daily routine (“extra” = taking stairs rather than the elevator, walking pets, parking further away in the parking lot, etc.)

______ Participate in sporting activity at least 1-2 times weekly (e.g., golf, volleyball, basketball, tennis)

______ Eat 5 or more servings of fruits and vegetables daily

______ Consistently choose foods with whole grains

______ Choose foods high in fiber

______ Lose or maintain body weight

______ Avoid smoking or tobacco use

______ Implementing strategies to help with lifestyle modifications

______ Live an overall healthy lifestyle


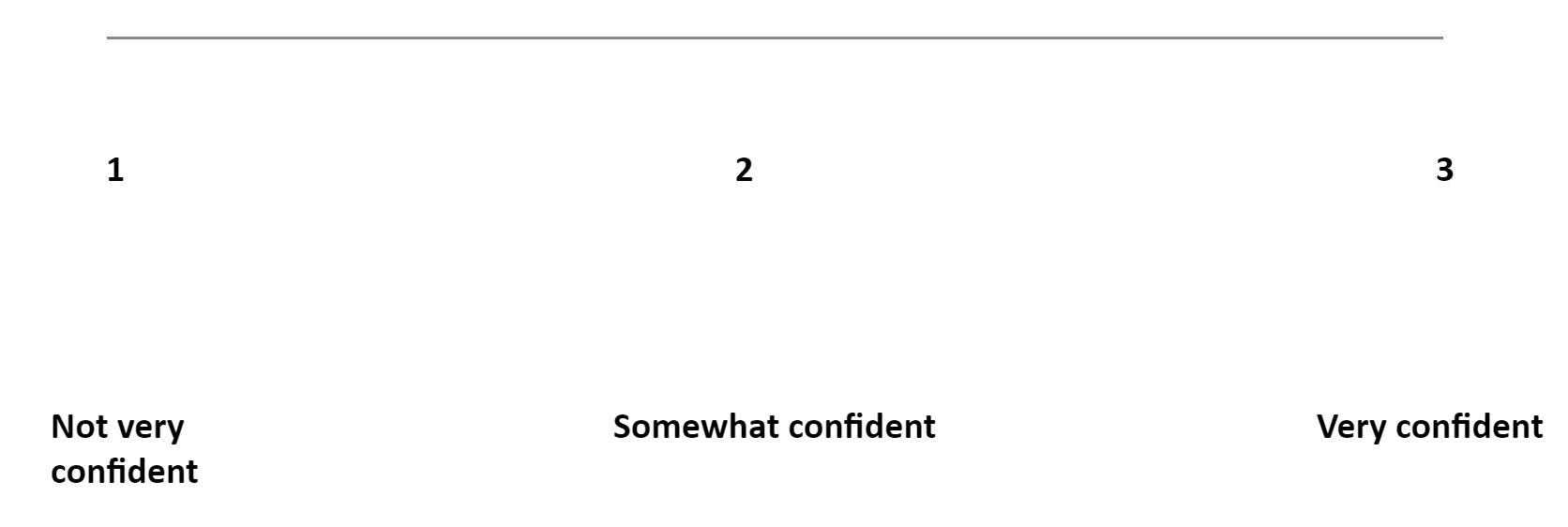


Confidence-to-Change Lifestyle Questionnaire

Name: _______________________________

Date: ________________________________

Rating Confidence-to-Change

3 Very confident

2 Somewhat confident

1 Not very confident

Rating Lifestyle behavior

______ Purposeful exercise at least 5 times per week

______ Incorporate “extra” physical activity throughout the daily routine (“extra” = taking stairs rather than the elevator, walking pets, parking further away in the parking lot, etc.)

______ Participate in sporting activity at least 1-2 times weekly (e.g., golf, volleyball, basketball, tennis)

______ Eat 5 or more servings of fruits and vegetables daily

______ Consistently choose foods with whole grains

______ Choose foods high in fiber

______ Lose or maintain body weight

______ Avoid smoking or tobacco use

______ Implementing strategies to help with lifestyle modifications

______ Live an overall healthy lifestyle

Continue immediately to the next set of questions (PSQI)

The Pittsburgh Sleep Quality Index (PSQI)

1. When do you usually go to bed? ______________
2. How long (in minutes) has it taken you to fall asleep each night? ________________
3. When have you usually gotten up in the morning? ______________
4. How many hours of actual sleep do you get at night? (This may be different than the number of hours you spend in bed) _____________________

| 5. During the past month, how often have you had trouble sleeping because you… | Not during the past month (0) | Less than once a week (1) | Once or twice a week (2) | Three or more times a week (3) |
| --- | --- | --- | --- | --- |
| 1. Cannot get to sleep within 30 minutes |  |  |  |  |
| 1. Wake up in the middle of the night or early morning |  |  |  |  |
| 1. Have to get up to use the bathroom |  |  |  |  |
| 1. Cannot breathe comfortably |  |  |  |  |
| 1. Cough or snore loudly |  |  |  |  |
| 1. Feel too cold |  |  |  |  |
| 1. Feel to hot |  |  |  |  |
| 1. Have bad dreams |  |  |  |  |
| 1. Have pain |  |  |  |  |
| 1. Other reason(s), please describe, including how often you have had trouble sleeping because of this reason(s): |  |  |  |  |
| 6. During the past month, how often have you taken medicine (prescribed or “over the counter”) to help you sleep? |  |  |  |  |
| 7. During the past month, how often have you had trouble staying awake while driving, eating meals, or engaging in social activity? |  |  |  |  |
| 8. During the past month, how much of a problem has it been for you to keep up enthusiasm to get things done? |  |  |  |  |
|  | Very good (0) | Fairly good (1) | Fairly bad (2) | Very bad (3) |
| 9. How would you rate your sleep quality over the past month? |  |  |  |  |

*After each participant completes the survey, the research associate scores the Pittsburgh Sleep Quality Index questionnaire and enters the score in REDCap.

**Total Sleep Index Score: ____ (Range 0 to 21)**

Do you currently smoke? □ Yes □ No Did you previously smoke? □ Yes □ No

If yes to either, how many packs per day (PPD) and how many years?
PPD: ____ Years: ____ (Maximum = 99)

Do you plan to make any lifestyle changes as a result of today’s visit? If so, what kind? Check all that apply.

❑ No planned changes

❑ Initiating a change in my diet

❑ Initiating or increasing my exercise in a safe manner

❑ Improving my sleep habits and/or hours of daily sleep

❑ Stop smoking

❑ Other __________________________________________________________________

**Select questions from the US Governmental HCAHPS Patient Satisfaction Survey**

During this hospital stay, how often did doctors treat you with courtesy and respect?

- Never
- Sometimes
- Usually
- Always

During this hospital stay, how often did doctors listen carefully to you?

- Never
- Sometimes
- Usually
- Always

During this hospital stay, how often did doctors explain things in a way you could understand?

- Never
- Sometimes
- Usually
- Always

Using any number from 0 to 10, where 0 is the worst hospital possible, and 10 is the best hospital possible, what number would you use to rate this hospital during your stay?

- 0 Worst hospital possible
- 1
- 2
- 3
- 4
- 5
- 6
- 7
- 8
- 9
- 10 Best hospital possible

Would you recommend this hospital to your friends and family

- Definitely no
- Probably no
- Probably yes
- Definitely yes

When I left the hospital, I had a good understanding of the things I was responsible for in managing my health.

- Strongly disagree
- Disagree
- Agree
- Strongly agree

In general, how would you rate your overall health?

- Excellent
- Very good
- Good
- Fair
- Poor

What is the highest grade or level of school that you have completed?

- 8th grade or less
- Some high school but did not graduate
- Good
- High school graduate or GED
- Some college or 2-year degree
- 4-year college graduate
- More than a 4-year college degree

May we contact you for a follow-up after this visit? If so, what number? ___________________

How can we make this more effective in helping you make changes to prevent diseases in your life? (If randomized to the video, ask how the video can be improved.) ____________________________________________________________________________________________________________________________________________________________
