# Supplementary material for: Brief interactive lifestyle preventive medicine video education in the primary care clinic: Protocol for a randomized clinical trial
Source: PLoS One. 2026 Mar 26;21(3):e0322244. doi: 10.1371/journal.pone.0322244 (PMC13020839; doi:10.1371/journal.pone.0322244)
Supplement: S3 Appendix — Follow-up survey and script. (DOCX) [file pone.0322244.s003.docx]

**S3 appendix C: follow-up survey and script**

Hello! Is this (Patient Name)?

This is (insert research associate name); I’m part of the research team. You consented at your primary care clinic visit on (insert clinic visit date) that we could call you at this number for a follow-up survey regarding your lifestyle behaviors. Are you free to answer our survey of a few questions?

If no:

That is okay! When would be a good time for us to call you back for a follow-up?

Date/Time: ____________

If yes:

Have you adopted any of the lifestyle changes discussed in the video shown to you in the clinic regarding diet, sleep, exercise, or smoking?

If so, what changes have you adopted?

Those are all the questions! Thank you so much for your time and have a great day!

**Follow-up patient interview - prior to interview:**

**List diagnoses from the last two office visits:**

**______________________________________________________________________________ ______________________________________________________________________________ ______________________________________________________________________________**

Is chest pain included as one of the above diagnoses? ❑ No ❑ Yes ❑ Don’t Know

**Interview:** Please select the party providing the information for this interview. Check all that apply (may include more than one person present during the interview). **Note:** only patients willing and able to participate in the original interview will complete the survey. However, some patients may request another person to answer the questionnaires. Please indicate here who provided answers to the questionnaires:

❑ Patient ❑ Spouse/Significant Other ❑ Sibling ❑ Child ❑ Other Family Member ❑ Medical Care Provider ❑ Other: _________________________

1. Did the patient receive follow-up care after their index PCP visit?

❑ No ❑ Yes ❑ Don’t Know

2. Is the patient doing well?

❑ No ❑ Yes ❑ Don’t Know

3. Have you adopted any lifestyle changes, including diet, exercise, sleep, or smoking?

❑ No ❑ Yes ❑ Don’t Know

Specify here which changes: ________________________________________________________________________________________________________________________________________________

Note any special considerations here, e.g. patient in hospital now, at a follow-up appointment during the time of call, the patient is deceased:

________________________________________________________________________
