# Supplementary material for: Brief interactive lifestyle preventive medicine video education in the primary care clinic: Protocol for a randomized clinical trial
Source: PLoS One. 2026 Mar 26;21(3):e0322244. doi: 10.1371/journal.pone.0322244 (PMC13020839; doi:10.1371/journal.pone.0322244)

**S4 appendix D: educational video used for prevention and script**

If informed consent is obtained, proceed with randomization of video or no video.

If the patient is randomized to video, tell the patient: “Your provider (optional - give the name of provider here) would like you to watch a video that will teach you how to live a healthy lifestyle. Would you like to watch it now?”

Link for Video Playlist:
https://h5p.org/node/1412622


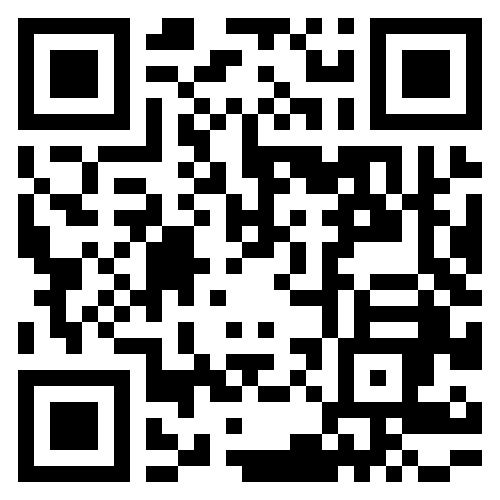

Supplement: S4 Appendix — Educational video used for prevention and script. (DOCX) [file pone.0322244.s004.docx]
